# Supplementary material for: Adjunctive Bright Light Therapy for Non-Seasonal Major Depressive Disorder: A Randomized Controlled Trial
Source: Int J Environ Res Public Health. 2022 Sep 29;19(19):12430. doi: 10.3390/ijerph191912430 (PMC9566126; doi:10.3390/ijerph191912430)
Supplement: Supplementary file 1 [file ijerph-19-12430-s001.zip › ijerph-1895977-supplementary-for correction.pdf]

Supplementary Materials

Table S1. The subscales of HAMD-17 and their items

Number	HAMD-17 items	Bech-6	Maier-6	Gibbons-8	Santen-7	Anxiety-6	Retardation-4
1	Depressed mood	•	•	•	•		•
2	Feelings of guilt	•	•	•	•		
3	Suicide			•	•		
4	Insomnia-early						
5	Insomnia-middle						
6	Insomnia-late						
7	Work and activities	•	•	•	•		•
8	Retardation	•	•		•		•
9	Agitation		•	•			
10	Anxiety-psychic	•	•	•	•	•	
11	Anxiety-somatic			•		•	
12	Somatic symptoms-gastrointestinal					•	
13	Somatic symptoms-general	•			•	•	
14	Genital symptoms			•			•
15	Hypochondriasis					•	
16	Loss of weight						
17	Loss of insight					•	

HAMD-17: Hamilton Depression Rating Scale-17 items; Bech: Bech melancholia scale; Maier: Maier–Philipp severity subscale; Gibbons: Gibbons global depression severity scale; Santen: Santen scale; Anxiety: anxiety subscale; Retardation: retardation subscale.

Table S2. Outcome measures (subscales of HAMD-17) in treating major depressive disorder over time using bright light therapy (n = 22) and dim red-light (n = 21)

		**Total score**		***p***	**Change of score**		***p***	**Response**		***p***
		**BLT**	**DRL**		**BLT**	**DRL**		**BLT**	**DRL**	
**Outcome**	**Week**	**Mean (SD)**	**Mean (SD)**		**Mean (SD)**	**Mean (SD)**		***n* (%)**	***n* (%)**	
**Bech**	0	10.23 (2.11)	9.67 (2.37)	0.42						
	1	9.23 (2.51)	7.86 (2.97)	0.11	1.00 (1.72)	1.81 (2.80)	0.26	1 (4.50)	1 (4.80)	1.00
	2	7.86 (2.85)	7.57 (2.96)	0.74	2.36 (2.74)	2.10 (3.00)	0.76	4 (18.2)	4 (19.0)	1.00
	4	7.00 (3.27)	6.62 (3.34)	0.71	3.23 (2.83)	3.05 (4.21)	0.87	8 (36.4)	9 (42.9)	0.66
**Maier**	0	9.73 (2.43)	9.05 (2.77)	0.40						
	1	8.82 (2.75)	7.38 (3.01)	0.11	0.91 (2.05)	1.67 (2.96)	0.33	1 (4.5)	1 (4.8)	0.97
	2	7.32 (2.90)	7.29 (3.45)	0.97	2.41 (3.10)	1.76 (3.36)	0.52	4 (18.2)	3 (14.3)	1.00
	4	6.55 (3.08)	6.19 (3.67)	0.73	3.18 (3.08)	2.86 (4.51)	0.78	9 (40.9)	10 (47.6)	0.66
**Gibbons**	0	13.00 (3.41)	12.00 (4.07)	0.39						
	1	11.32 (3.70)	9.29 (4.38)	0.11	1.68 (2.89)	2.71 (3.77)	0.32	2 (9.1)	2 (9.5)	1.00
	2	9.73 (3.68)	8.81 (4.83)	0.49	3.27 (4.09)	3.19 (4.23)	0.95	5 (22.7)	6 (28.6)	0.66
	4	8.64 (3.89)	8.29 (5.35)	0.81	4.36 (3.75)	3.71 (5.92)	0.67	9 (40.9)	8 (38.1)	0.85
**Santen**	0	12 (2.64)	11.43 (2.73)	0.49						
	1	10.36 (3.03)	8.95 (3.46)	0.16	1.64 (1.97)	2.48 (3.36)	0.32	1 (4.5)	1 (4.8)	1.00
	2	8.95 (3.05)	8.57 (3.57)	0.71	3.05 (3.27)	2.86 (3.58)	0.86	4 (18.2)	4 (19.0)	0.94
	4	8.00 (3.87)	7.90 (4.00)	0.94	4.00 (3.52)	3.52 (4.90)	0.72	7 (31.8)	9 (42.9)	0.45

**Anxiety**	0	6.95 (1.82)	6.33 (2.44)	0.35						
	1	6.00 (1.75)	5.05 (2.27)	0.13	0.95 (1.73)	1.29 (1.77)	0.54	1 (4.50)	2 (9.50)	0.61
	2	5.32 (2.12)	4.43 (2.48)	0.21	1.64 (2.13)	1.90 (1.58)	0.64	4 (18.20)	6 (28.6)	0.49
	4	4.77 (2.33)	4.67 (2.87)	0.90	2.18 (2.06)	1.67 (2.13)	0.43	7 (31.8)	7 (33.3)	0.92
**Retardation**	0	6.45 (1.71)	6.24 (1.76)	0.69						
	1	6.05 (1.70)	5.24 (2.00)	0.16	0.41 (1.18)	1.00 (1.95)	0.23	0 (0)	3 (14.3)	0.11
	2	5.27 (2.03)	5.33 (1.74)	0.72	1.18 (1.89)	0.90 (1.95)	0.64	4 (18.2)	2 (9.5)	0.66
	4	4.73 (2.31)	4.67 (2.08)	0.93	1.73 (2.43)	1.57 (2.84)	0.85	7 (31.8)	6 (28.6)	0.82

HAMD-17: Hamilton Depression Rating Scale-17 items; Bech: Bech melancholia scale; Maier: Maier–Philipp severity subscale; Gibbons: Gibbons global depression severity scale; Santen: Santen scale; Anxiety: anxiety subscale; Retardation: retardation subscale; SD: standard deviation.

Table S3. Comparison of the effects on subscales of HAMD-17 in treating major depressive disorder for 4 weeks between bright light therapy and dim red-light groups, using hierarchical linear mixed-modeling[a] to show group effect, time effect, and interaction of group and time

	Group effect			Time effect			Group × time effect		
**Outcome**	***B***	**95% CI**	***p***	***B***	**95% CI**	***p***	***B***	**95% CI**	***p***
**Bech**	1.22	−0.40 to 2.8	0.14	−0.62	−0.03 to −0.20	0.04	−0.49	−1.31 to 0.32	0.23
**Maier**	1.20	−0.56 to 2.96	0.18	−0.60	−1.21 to 0.02	0.06	−0.54	−1.40 to 0.31	0.21
**Gibbons**	2.17	−0.21 to 4.56	0.07	−0.50	−1.28 to 0.28	0.20	−0.84	−1.93 to 0.24	0.13
**Santen**	1.53	−0.40 to 3.47	0.12	−0.52	−1.22 to 0.17	0.14	−0.66	−1.63 to 0.31	0.18
**Anxiety**	1.19	−0.09 to 2.48	0.07	−0.19	−0.63 to 0.25	0.39	−0.42	−1.04 to 0.19	0.18
**Retardation**	0.79	−0.26 to 1.84	0.14	−0.29	−0.69 to 0.12	0.16	−0.37	−0.94 to 0.19	0.19

HAMD-17: Hamilton Depression Rating Scale-17 items; Bech: Bech melancholia scale; Maier: Maier–Philipp severity subscale; Gibbons: Gibbons global depression severity scale; Santen: Santen scale; Anxiety: anxiety subscale; Retardation: retardation subscale; 95% CI: 95% confidence interval; [a]Adjusted for age, gender, and baseline scores.
